# Supplementary material for: Anticoagulation with Factor Xa Inhibitors Is Associated with Improved Overall Response and Progression-Free Survival in Patients with Metastatic Malignant Melanoma Receiving Immune Checkpoint Inhibitors—A Retrospective, Real-World Cohort Study
Source: Cancers (Basel). 2021 Oct 12;13(20):5103. doi: 10.3390/cancers13205103 (PMC8534137; doi:10.3390/cancers13205103)
Supplement: Supplementary file 1 [file cancers-13-05103-s001.zip › cancers-1378273-supplementary_rev_MH_111021.pdf]

cancers

MDPI

# Supplementary Figures and Tables

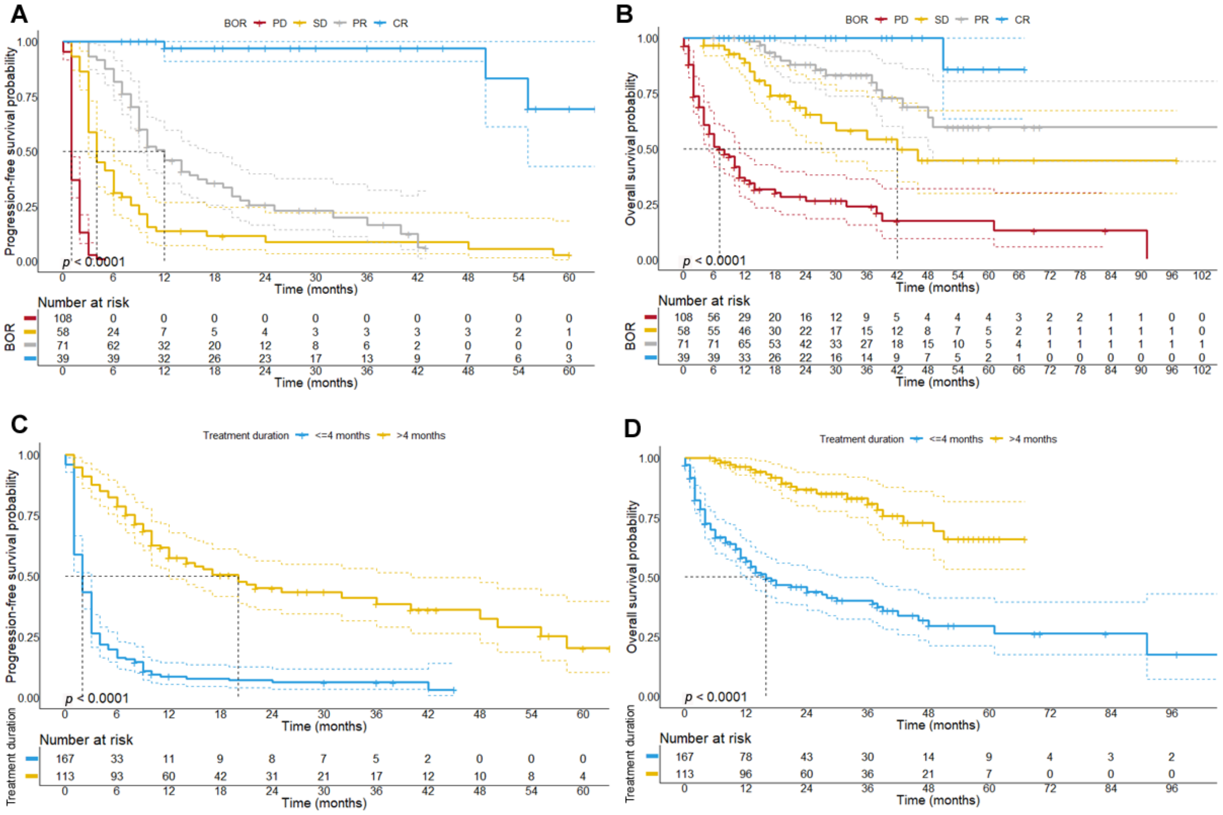


**Supplementary Figure S1: Kaplan Meier plots showing the progression-free (left) and overall survival probabilities of melanoma patients stratified by the BOR to initial ICI (top) and the duration of initial ICI therapy (bottom).** Consistent with previous studies BOR has been the best predictor of PFS (median PFS: 0 vs 4 vs 12 months vs not reached, $p$<0.0001) (**A**) and OS (median OS: 7 vs 42 months vs not reached for PR and CR, $p$<0.0001) (**B**). It has further been found that patients remaining on initial ICI therapy for at least 5 months had a significantly longer PFS (1 month; 95% CI: 0 – 2.0 months, vs 20 months; 95% CI: 12.2 – 27.8 months; $p$<0.0001) (**C**) and OS (16 months vs not reached, $p$<0.0001) (**D**) as compared to patients receiving initial ICI therapy for less than 5 months.

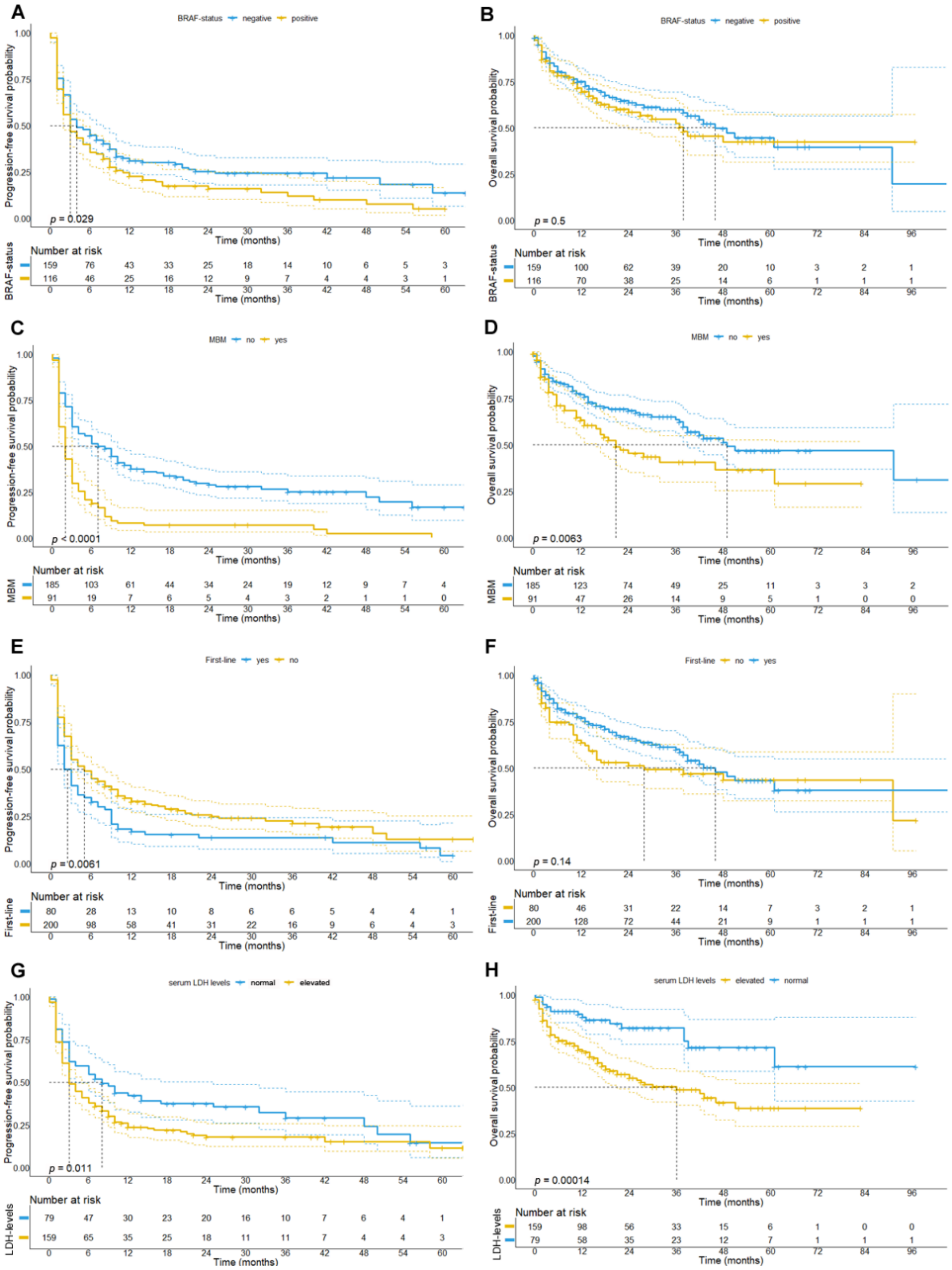


**Supplementary Figure S2: Kaplan Meier plots showing the progression-free (left) and overall survival probabilities (right) for melanoma patients stratified by BRAF-status (A,B), the presence of MBM (C,D), the administration of ICI in a first-line setting (E,F) and LDH-serum-levels (G,H).** Patients with BRAF-mutated melanoma showed a worse PFS (median: 3 vs 4 months; $p$=0.032), but not OS (median: 38 vs 46 months; p=0.504) as compared to patients with BRAF-wildtype melanoma. Patients not developing MBM showed a significantly better PFS (median: 7 vs 1 months; $p$<0.0001) and OS (median: 21 vs 49 months; p=0.006) as compared to patients with MBM. Moreover, patients receiving ICI therapy in a first-line setting showed a significantly better PFS (median: 5 vs 2 months, $p$=0.006), but not OS (median: 46 vs 28 months, $p$=0.14) as compared to patients given ICI after systemic pretreatments. Notably, patients with normal serum LDH-levels (<245 U/l) showed a better PFS (median: 8 vs 3 months, $p$=0.01) and OS (median: not reached vs 36 months, $p$<0.0001) as compared to patients with elevated LDH-levels at ICI initiation.

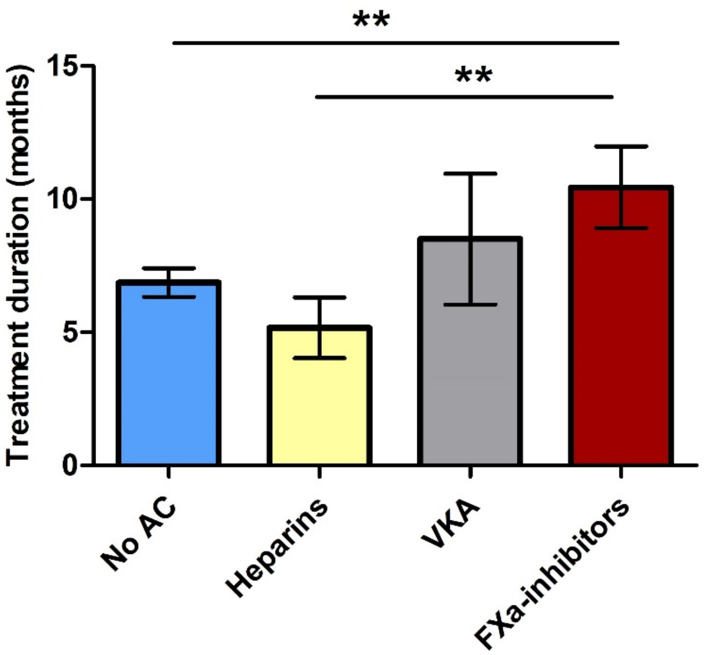


**Supplementary Figure S3: Treatment duration of initial ICI therapy stratified by the different categories of concomitant AC.** Patients receiving concomitant FXa-inhibitors remained significantly longer on initial ICI therapy (mean: 10.4 months; 95% CI: 7.3-13.6 months) as compared to patients not receiving AC (mean: 6.9 months; 95% CI: 5.8-7.9 months) or those with concomitant heparin treatment (mean: 5.2 months; 95% CI: 2.8-7.5 months), whereas patients receiving VKA were heterogeneous in terms of ICI treatment duration (mean: 8.5 months; 95% CI: 3.4-13.6 months). When comparing patients with concomitant FXa-inhibitor therapy to all other subgroups combined, this association was below statistical significance ($p$=0.063). *Abbreviations: *p<0,05, **p<0,005, ***p<0,001.*

**Supplementary Table S1: Univariate Cox proportional hazards model for progression-free survival**

| Variables | Subgroups | HR | 95% CI | *p*-value |
|---|---|---|---|---|
| Age (years) | >66 vs ≤66 | 0.91 | 0.69-1.19 | 0.47 |
| Gender | Female vs male | 0.94 | 0.72-1.23 | 0.65 |
| LDH | Elevated vs normal | 1.51 | 1.1-2.08 | **0.011** |
| Ulceration | No vs Yes | 0.87 | 0.62-1.2 | 0.42 |
| Breslow (mm) | ≤2mm vs >2mm | 0.89 | 0.65-1.22 | 0.46 |
| AC-status | No vs Yes | 0.95 | 0.71-1.28 | 0.73 |
| AC-category | FXa-i vs other | 0.48 | 0.28-0.81 | **0.006** |
| BRAF status | Pos vs Neg | 1.33 | 1.02-1.74 | **0.036** |
| Brain metastases | Yes vs no | 2.39 | 1.81-3.16 | **<0.001** |
| Pretreatments | Yes vs no | 1.17 | 0.89-1.52 | 0.26 |
| Line of treatment | First vs other | 0.67 | 0.5-0.89 | **0.0056** |
| BOR to initial ICI | CR, PR vs SD,PD | 0.14 | 0.10-0.19 | **<0.001** |
| Treatment duration (months) | >4 vs ≤4 | 0.21 | 0.15-0.29 | **<0.001** |
| Bleeding complications | No vs Yes | 0.57 | 0.36-0.90 | **0.016** |

The p value is indicated in bold numbers when statistically significant. Abbreviations: AC = anticoagulation, FXai = Factor Xa-inhibitors, CR = complete response, PR = partial response, SD = stable disease, PD = Progressive disease, BOR = best overall response, HR = hazard ratio, CI = confidence interval

**Supplementary Table S2: Univariate Cox proportional hazards model for overall survival**

| Variables | Subgroups | HR | 95% CI | *p*-value |
|---|---|---|---|---|
| Age (years) | >66 vs ≤66 | 1.03 | 0.71-1.50 | 0.88 |
| Gender | Female vs male | 0.74 | 0.49-1.09 | 0.13 |
| LDH | Elevated vs normal | 2.74 | 1.59-4.71 | **<0.001** |
| Ulceration | Yes vs No | 0.91 | 0.56-1.46 | 0.68 |
| Breslow (mm) | >2 vs ≤2 | 0.88 | 0.65-1.21 | 0.46 |
| AC-status | No vs Yes | 0.81 | 0.54-1.22 | 0.32 |
| AC-category | FXa-i vs other | 0.49 | 0.22-1.12 | 0.09 |
| BRAF status | Pos vs Neg | 1.14 | 0.78-1.65 | 0.49 |
| Brain metastases | Yes vs no | 1.69 | 1.16-2.45 | **0.007** |
| Pretreatments | Yes vs no | 1.07 | 0.73-1.55 | 0.73 |
| Line of treatment | First vs other | 0.74 | 0.51-1.1 | 0.13 |
| BOR to initial ICI | CR, PR vs SD,PD | 0.15 | 0.09-0.25 | **<0.001** |
| Treatment duration (months) | >4 vs ≤4 | 0.20 | 0.12-0.32 | **<0.001** |
| Post-ICI treatment lines | =>1 vs 0 | 0.67 | 0.43-1.04 | 0.074 |
| Bleeding complications | No vs Yes | 0.42 | 0.25-0.71 | **0.001** |

The p value is indicated in bold numbers when statistically significant. Abbreviations: AC = anticoagulation, FXai = Factor Xa-inhibitors, CR = complete response, PR = partial response, SD = stable disease, PD = Progressive disease, BOR = best overall response, HR = hazard ratio, CI = confidence interval

**Supplementary Table S3: Multivariate Cox proportional hazards model for progression-free survival.**

| Variables | Subgroups | HR | 95% CI | *p*-value |
|---|---|---|---|---|
| LDH | Elevated vs normal | 1.43 | 1.03-1.99 | **0.035** |
| BRAF status | Pos vs Neg | 1.25 | 0.90-1.74 | 0.192 |
| Brain metastases | Yes vs No | 2.26 | 1.62-3.14 | **<0.001** |
| Treatment duration (months) | >4 vs ≤4 | 0.23 | 0.16-0.33 | **0.003** |
| BOR to initial ICI | CR, PR vs SD, PD | 0.19 | 0.11-0.33 | **<0.001** |
| AC-category | FXa-i vs other | 0.53 | 0.27-0.81 | **0.007** |

The p value is indicated in bold numbers when statistically significant. Abbreviations: AC = anticoagulation, FXai = Factor Xa-inhibitors, CR = complete response, PR = partial response, SD = stable disease, PD = Progressive disease, BOR = best overall response, HR = hazard ratio, CI = confidence interval

**Supplementary Table S4: Multivariate Cox proportional hazards model for overall survival.**

| Variables | Subgroups | HR | 95% CI | *p*-value |
|---|---|---|---|---|
| LDH | Elevated vs normal | 2.18 | 1.23-3.87 | **0.008** |
| Brain metastases | Yes vs No | 1.95 | 1.18-3.24 | **0.01** |
| AC-category | FXa-i vs other | 0.77 | 0.32-1.81 | 0.55 |
| Treatment duration (months) | >4 vs ≤4 | 0.28 | 0.14-0.56 | **<0.001** |
| BOR to initial ICI | CR, PR vs SD, PD | 0.17 | 0.10-0.31 | **<0.001** |
| Post-ICI treatment lines | 0 vs >=1 | 0.25 | 0.15-0.44 | **<0.001** |
| Bleeding complications | No vs Yes | 0.52 | 0.28-0.97 | **0.04** |

The p value is indicated in bold numbers when statistically significant. Abbreviations: AC = anticoagulation, FXai = Factor Xa-inhibitors, CR = complete response, PR = partial response, SD = stable disease, PD = Progressive disease, BOR = best overall response, HR = hazard ratio, CI = confidence interval

**Supplementary Table S5: Patient demographics, tumor characteristics and specifics on concomitant AC treatment stratified by the class of AC received during initial ICI.** Chi-square test and ANOVA variance analysis revealed no significant differences between the different subcohorts, except for patients given FXa-inhibitors presented with thicker melanomas at initial diagnosis and patients on concomitant heparin treatment less often received initial ICI in a first-line setting.

| | **Heparins** | **Vit-K Antagonists** | **FXa inhibitors** | *p*-value |
|---|---|---|---|---|
| Reasons for AC[1] | | | | 0.34 |
| - DVT | 11 | 3 | 9 | |
| - PE | 6 | 12 | 4 | |
| - AF | 1 | 2 | 13 | |
| - Other | 11 | 3 | 1 | |
| Thromboembolic events during ICI | 13 (59.3%) | 3 (40%) | 9 (31.0%) | 0.095 |
| PTT | 29.2 | 33.8 | 30.4 | **0.04** |
| Mean Age at ICI initiation (years) | 64.1 | 70.5 | 71.0 | 0.13 |
| Gender (female) | 13 (44.8%) | 5 (25.0%) | 11 (40.7%) | 0.35 |
| Mean Breslow thickness (mm) | 2.1 | 3.4 | 3.4 | **0.04** |
| Ulceration | 8 (27.6%) | 10 (50.0%) | 10 (37.0%) | 0.70 |
| BRAF-mutation | 12 (41.4%) | 7 (35.0%) | 9 (33.3%) | 0.83 |
| LDH | 385.8 | 322.7 | 382.0 | 0.75 |
| Brain metastases | 12 (41.4%) | 4 (20.0%) | 10 (37.0%) | 0.27 |
| Pre-treatments | 14 (48.3%) | 6 (30.0%) | 8 (29.6%) | 0.27 |
| First | 16 (55.2%) | 16 (80.0%) | 23 (85.2%) | **0.03** |
| Class of ICI (cICB) | 7 (24.1%) | 4 (20.0%) | 9 (33.3%) | 0.61 |
| Post-treatment lines | 22 (75.9%) | 10 (50.0%) | 11 (40.7%) | 0.097 |
| ICI-Rechallange | 13 (44.8%) | 9 (45.0%) | 7 (29.6%) | 0.44 |

[1] Reasons for anticoagulation as documented in the medical chart review. For n=7 patients no data on the reasons for AC have been available. The p value is indicated in bold numbers when statistically significant. Abbreviations: DVT= deep vein thrombosis, AF= atrial fibrillation; PE= pulmonary embolism; ICI = immune-checkpoint-inhibitor(s); cICB = combined immune-checkpoint blockade.

**Supplementary Table S6: Status of ICI treatment at the time of data cut-off and reasons for treatment discontinuation stratified by the different AC categories.**

| | No AC | heparins | Vit-K Antagonists | FXa inhibitors |
|---|---|---|---|---|
| Ongoing | 13 (6.4%) | 0 | 2 (10%) | 6 (21.4%) |
| Cessation due to toxicity | 51 (25.0%) | 5 (17.3%) | 3 (15%) | 8 (28.6%) |
| Cessation due to progression | 80 (39.4%) | 16 (59.3%) | 8 (40%) | 4 (14.8%) |
| Other reasons[1] | 59 (28.9%) | 8 (27.6%) | 7 (35%) | 10 (37.0%) |

*Abbreviations*: [1] Other reasons include wish of the patient, ongoing subclinical toxicity or regular treatment cessation upon long-term complete response
