# Supplementary figures and images for: Anticoagulation with Factor Xa Inhibitors Is Associated with Improved Overall Response and Progression-Free Survival in Patients with Metastatic Malignant Melanoma Receiving Immune Checkpoint Inhibitors—A Retrospective, Real-World Cohort Study
Source: Cancers (Basel). 2021 Oct 12;13(20):5103. doi: 10.3390/cancers13205103 (PMC8534137; doi:10.3390/cancers13205103)

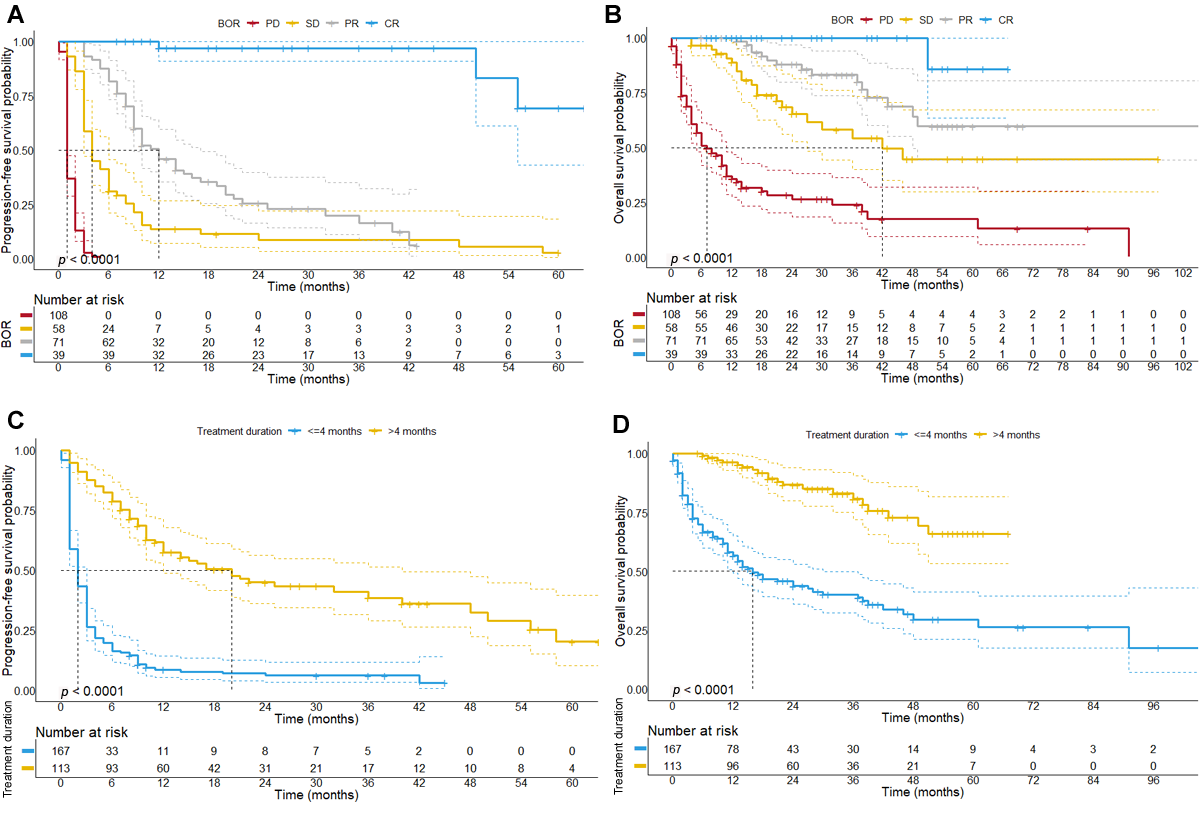

Supplement: Supplementary file 1 [file cancers-13-05103-s001.zip › Supplementary_Figure_1_revised_111021.tif]

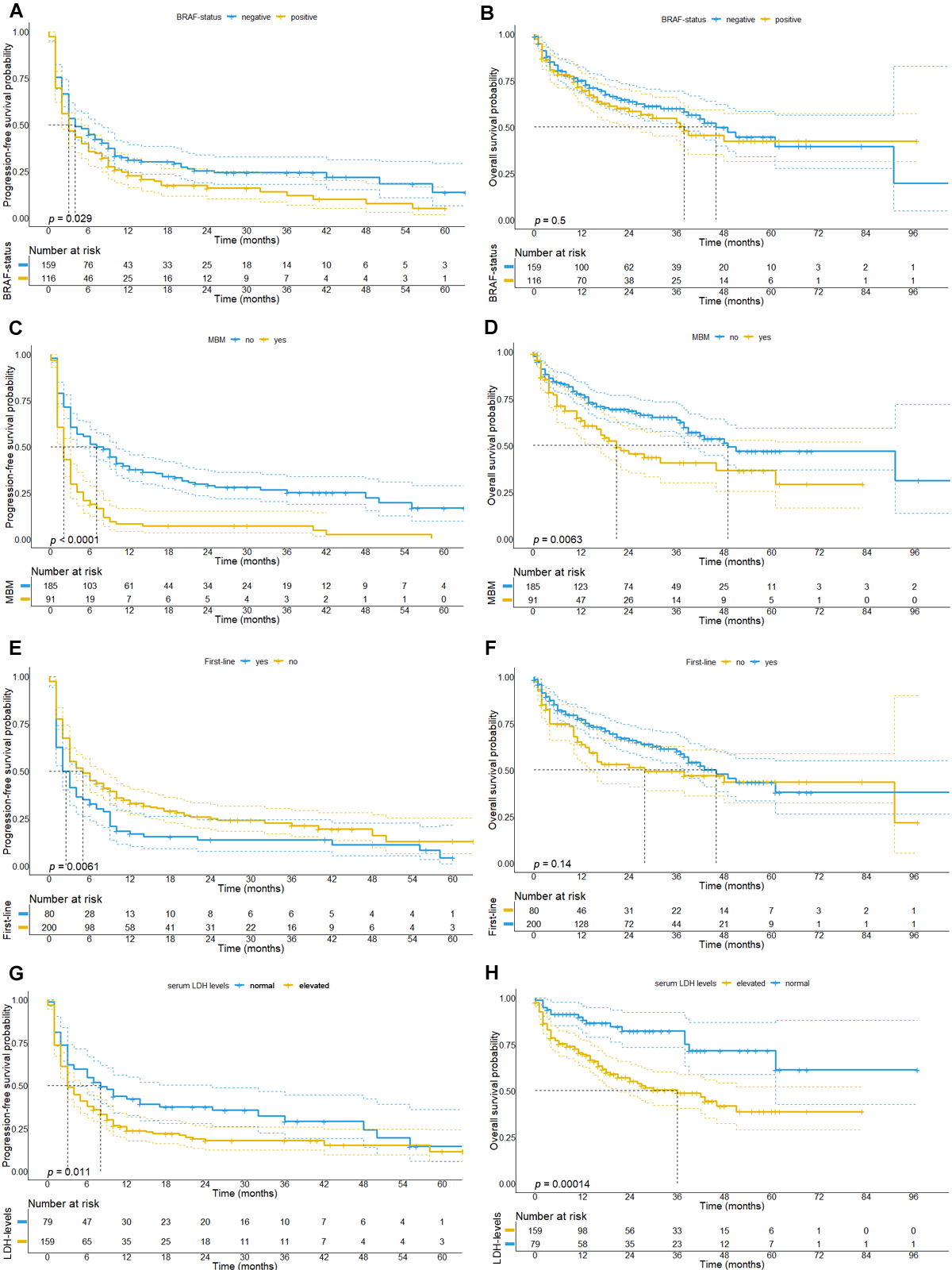

Supplement: Supplementary file 1 [file cancers-13-05103-s001.zip › Supplementary_Figure_2_revised_111021.tif]
